# Illegitimate Tasks and Nurses’ Workplace Well-Being: The Mediating Role of Emotional Exhaustion and the Moderating Role of Victim Justice Sensitivity

**DOI:** 10.3390/healthcare14142132

**Published:** 2026-07-16

**Authors:** Hongchi Guo, Po-Chien Chang

**Affiliations:** School of Business, Macau University of Science and Technology, Macau 999078, China; 3240004775@student.must.edu.mo

**Keywords:** illegitimate tasks, workplace well-being, emotional exhaustion, victim justice sensitivity

## Abstract

**Background/Objectives**: In healthcare settings, nurses frequently face illegitimate tasks that may threaten their professional identity and weaken their workplace well-being. Prior research drawing on conservation of resources (COR) theory has largely explained illegitimate tasks in terms of resource loss, whereas less attention has been given to these tasks from a justice theory perspective. To address this gap, the objective of this study was to examine how illegitimate tasks are associated with nurses’ workplace well-being by integrating COR theory and justice theory. Specifically, emotional exhaustion was examined as a mediator, and victim justice sensitivity as a moderator. **Methods**: Data were collected from nurses across multiple hospitals in China using a time-lagged design. After matching responses across the two waves, 497 valid cases were retained for the final analysis. The hypothesized moderated mediation model was tested using Hayes PROCESS Model 7, with 5000 bootstrap samples. **Results**: Illegitimate tasks were negatively associated with workplace well-being (B = −0.359, *p* < 0.001). Emotional exhaustion partially mediated this association (indirect effect = −0.186, 95% CI [−0.239, −0.137]). Victim justice sensitivity significantly moderated the association between illegitimate tasks and emotional exhaustion (B = 0.214, *p* < 0.001). Furthermore, the moderated mediation index was significant (index = −0.090, 95% CI [−0.121, −0.060]), indicating that the indirect association became stronger at higher levels of victim justice sensitivity. **Conclusions**: The findings indicate that illegitimate tasks are associated with lower workplace well-being among nurses, with this association partially explained by emotional exhaustion, particularly among nurses with high victim justice sensitivity. By integrating COR theory and justice theory, the findings highlight the dual burden of resource depletion and unfairness perceptions. Healthcare organizations should minimize illegitimate tasks to protect nurses’ workplace well-being.

## 1. Introduction

The well-being of nurses, as a core workforce responsible for patient care in the healthcare system, is closely related not only to their own health and job performance but also to the overall quality of patient care [1,2]. When nurses experience high levels of workplace well-being (WWB), they are generally more likely to perform their work duties with a more positive attitude and greater efficiency [3]; however, most existing studies on nurses’ well-being have considered it as a holistic psychological or professional state, paying little attention to WWB. Compared with general well-being, WWB more directly reflects individuals’ subjective evaluations and emotional responses in relation to their work roles, task performance, and organizational interactions, and it has direct implications for their work efficiency, health status, and overall quality of life [4]; therefore, a specific focus on nurses’ WWB is of urgent practical importance. Accordingly, this study adopts nurses’ WWB as the core outcome variable. Specifically, Zheng’s (2015) definition of WWB is adopted, which views well-being as the combination of job satisfaction and work-related emotional experiences formed by individuals in the workplace [5].

Moreover, previous studies on nurses’ WWB have paid little attention to special stressors related to role violation and identity threat. Most existing studies have mainly focused on general stressors, such as heavy workloads [6], inadequate staffing [7], and poor physical work environments [8]. Illegitimate tasks (ILTAs), as a specific stressor that violates role expectations and threatens individuals’ professional identity [9], can have negative effects on individuals’ cognition, emotions, and behaviors [9,10,11,12,13,14,15,16]. Compared with many other occupational groups, nurses may be particularly vulnerable to ILTAs because nursing is a highly role-defined profession centered on direct patient care [17,18]. Nurses are expected to devote their professional expertise primarily to patient care, and assignments that fall outside their professional responsibilities may not only increase unnecessary workload but also interfere with core nursing activities and threaten their professional identity [17]. Furthermore, reductions in clerical and support staff within healthcare institutions have resulted in many supportive and administrative tasks being transferred to nurses, increasing their involvement in non-nursing activities [17]. In addition, nursing work is characterized by high emotional demands, heavy workloads, and limited opportunities to refuse additional assignments. Particularly in the Chinese context, where a culture of high power distance prevails, individuals often find it difficult to refuse tasks assigned by superiors that fall outside their job scope, even if they consider such work to be both unnecessary and unreasonable [19]. Therefore, ILTAs are a specific stressor because they can drain nurses’ resources, offend their work roles, and threaten their professional identity [9]. For this reason, ILTAs have received increasing attention in healthcare management and nursing research [17]. Existing research has shown that, as role-offending stressors, ILTAs are associated with emotional strain, lower well-being, and negative work-related outcomes [10]; however, how ILTAs influence nurses’ WWB remains insufficiently understood within the nursing context [19]. Moreover, previous studies have primarily explained the effects of ILTAs from the perspective of resource depletion [12], while paying relatively little attention to how fairness-related perceptions and individual differences shape nurses’ responses to these tasks. These research gaps highlight the need to further clarify the psychological mechanism and boundary conditions through which ILTAs are associated with nurses’ WWB.

According to COR theory, the effects of work-related stressors on individuals’ WWB do not occur immediately but, rather, unfold gradually through psychological processes [20]. When individuals perceive that their resources are subject to potential or actual loss, or when individuals perceive that their resource investment does not bring the expected returns, they may develop stress responses, which may further lead to psychological consequences such as emotional exhaustion (EE) and, ultimately, undermine their positive evaluations of and affective experiences at work [21]. Therefore, we introduce EE, a state of emotional overextension and depletion of emotional resources [22], to explain the underlying process linking ILTAs to nurses’ WWB.

In addition, based on justice theory, individuals’ responses to work-related stressors largely depend on their subjective perceptions of fairness and unfairness [22,23,24]. Justice theory further suggests that individuals who perceive themselves as being unfairly treated or deprived of due respect are likely to experience negative emotional reactions, which may subsequently influence their psychological states and behavioral responses [25]. On this basis, victim justice sensitivity (VJS), as an important personal trait, reflects individuals’ sensitivity to whether they are treated fairly in potentially unfair situations [21]. People with greater VJS tend to view ILTAs as unfair treatment, thereby amplifying their perceptions of resource loss and intensifying their psychological stress reactions [21,26], and be more protective of their existing resources. This study incorporates VJS as a moderator to examine whether the associations of ILTAs with EE and nurses’ WWB differ across levels of VJS.

Guided by COR theory and justice theory, this research explores the process through which ILTAs are associated with nurses’ WWB after they are required to perform such tasks. The analysis further identifies EE as the explanatory pathway and VJS as the condition under which this pathway varies. Accordingly, the objective of the present study was to examine the association between ILTAs and nurses’ WWB by investigating the mediating role of EE and the moderating role of VJS. In the nursing context, ILTAs differ from general job stressors such as heavy workload or role ambiguity; they are better understood as role-offending stressors, as they may challenge nurses’ professional role perceptions and thereby impair their WWB. A two-wave survey was conducted among nurses in Chinese hospitals, yielding a final matched sample of 497 nurses. The findings may inform healthcare management practices aimed at reducing ILTAs and promoting sustainable well-being among nursing staff, while also enriching the theoretical understanding of how stressors influence nurses’ WWB.

## 2. Hypothesis Development

### 2.1. ILTAs and WWB

In healthcare settings, the continuous expansion of nursing work, staffing shortages, and mismatches between task allocation and professional role boundaries make nurses vulnerable to ILTAs [27]. Generally, ILTAs are work duties that fall outside an individual’s role boundaries and are regarded as an offense to one’s occupational identity [9]. Tasks that deviate from nurses’ core responsibilities may be perceived as a devaluation of their professional identity and role value, thereby eliciting negative reactions [15]. As the alignment between the tasks undertaken by nurses and their professional roles is considered to be an important factor influencing their WWB [17], ILTAs may undermine nurses’ WWB by disrupting their perceptions of professional role congruence and role value. On the one hand, individuals may perceive such tasks as a waste of time and energy [28]; on the other hand, these tasks may blur individuals’ professional roles, fail to provide meaningful goals, and remain inconsistent with employees’ higher-order occupational goals, thereby weakening the extent to which individuals experience accomplishment and meaning after completing such tasks [29]. At the same time, such tasks may lead individuals to feel disrespected, under-recognized, and devalued in their role, while also creating a perceived imbalance between effort and reward, thereby reducing their sense of satisfaction [30]. Moreover, when individuals believe that ILTA assignments stem from their own lack of competence, they tend to experience shame; when they see such assignments as resulting from poorly managed interpersonal relationships, they are likely to display anger [31]. Taken together, ILTAs may reduce employees’ job satisfaction, increase their stress and negative emotions, and weaken their sense of work meaningfulness [10]. These factors constitute important components of WWB [5]; therefore, when nurses frequently undertake ILTAs, their WWB may be undermined. Accordingly, the present study proposes the following hypothesis:

**H1.**  *ILTAs are negatively related to WWB*.

### 2.2. Mediating Effect of EE

In nursing work, nurses’ psychological resources are essential; they can help nurses deal with complex work situations [32]. ILTAs may disrupt individuals’ perceptions of role expectations and threaten their professional identity, thereby intensifying perceived resource depletion and, ultimately, leading to EE [12,33,34]. Within the COR theory, stress is understood to occur when individuals are threatened with resource loss, lose valued resources, or do not obtain expected returns after investing resources [35]. While providing patient care, nurses are often exposed to situations involving mistreatment, verbal aggression, and conflicts with patients; such stressful encounters in themselves deplete nurses’ psychological resources [32]. On this basis, ILTAs further require nurses to devote their limited time, effort, and mental resources to work activities that deviate from their professional role expectations, thereby weakening their ability to protect existing resources, intensifying their sense of resource loss, and ultimately leading to EE [33]. Moreover, ILTAs fall outside individuals’ expected professional roles, pose a threat to occupational identity, and may leave them feeling offended or devalued. These experiences can trigger negative emotions, including anger, anxiety, or frustration [36]. Therefore, ILTAs may cause resource loss among nurses and further intensify their EE. Building on the above theoretical reasoning, the present study proposes the following hypothesis:

**H2.**  *ILTAs are positively related to EE*.

From a COR perspective, how individuals judge their remaining resources can strongly affect how they think, feel, and behave. When individuals have enough resources, they may use them to keep their positive state and gain more resources; in contrast, when individuals lack resources, they may reduce further investment to avoid losing more [35]. WWB captures employees’ general evaluation of their work and feelings toward it, with job satisfaction and work-related affect as its core manifestations [5]. Therefore, EE may seriously strain individuals’ emotional and psychological resources, leaving them less able to view their job in a favorable light or sustain positive feelings at work and, in turn, weakening their well-being in the workplace [37]. Specifically, EE may impair individuals’ well-being at work in two main ways: On the one hand, emotionally exhausted individuals become less willing to invest further resources in their work. Individuals experiencing EE often tend to adopt a strategy of minimal engagement to cope with work demands [38], reducing their emotional investment, trust, and commitment to the team, and they may ultimately fail to realize their professional potential [39]. These negative psychological cognitions make it difficult for nurses to experience job satisfaction in complex work environments, thereby reducing their WWB. On the other hand, the resources available to individuals for generating well-being are diminishing. On a psychological level, EE may make individuals more vulnerable to depressive and anxious symptoms, thereby reducing their work efficiency [40]. In addition, EE may undermine individuals’ self-confidence and dignity, leaving them with a sense of helplessness [41]. Overall, individuals experiencing EE often find it difficult to respond to task demands with constructive emotions [42]. Repeated negative emotional experiences at work may further undermine individuals’ WWB. Accordingly, the present study proposes the following hypothesis:

**H3.**  *EE is negatively related to WWB*.

Building on these hypotheses, ILTAs may disrupt nurses’ perceptions of role expectations and threaten their occupational identity, thereby gradually draining their personal resources and, eventually, giving rise to EE. Such exhaustion means that individuals have little emotional and mental energy left to deal with ongoing work demands; it may weaken nurses’ readiness to commit additional personal resources to their work and reduce the resources available for fulfilling their role responsibilities, ultimately making it difficult for them to experience well-being at work. Therefore, the present study proposes the following hypothesis:

**H4.**  *EE mediates the relationship between ILTAs and WWB*.

### 2.3. Moderating Effect of VJS

From the perspective of justice theory, ILTAs may be perceived as a form of organizational unfairness. Such perceptions of unfairness convey negative information about individuals’ social standing and the degree of respect they receive within the organization, thereby threatening their professional identity and sense of self-worth [16]. According to justice theory, individuals evaluate whether they are treated fairly within an organization by considering whether distributive outcomes are reasonable, whether decision-making procedures are fair, and whether they receive adequate explanations, dignity, and respect during interpersonal interactions in the implementation of decisions [25]. However, individuals with different personality traits may differ in how they perceive and interpret fairness-related information [10]. Individuals with higher VJS are more likely to interpret ambiguous work situations as unjust and, consequently, experience stronger negative emotional reactions [21,43]. Therefore, when nurses with higher VJS are required to undertake ILTAs that fall outside their expected professional roles, they are more likely to perceive themselves as targets of unfair treatment and consequently experience stronger negative emotions, such as anger [44]. Specifically, they may perceive such tasks not only as unfair distributive outcomes of work assignments, but also as evidence of unjust task allocation procedures or a lack of adequate explanations, dignity, and respect from supervisors during the task assignment process. In such situations, nurses are likely to feel that they have not been fully respected and may perceive threats to their social self-esteem and occupational identity [16]. These perceptions may further intensify their sense of unfairness, accelerate psychological resource depletion, and ultimately lead to higher levels of EE.

Moreover, individuals with higher VJS are more likely to attend to, interpret, and repeatedly ruminate on unfairness-related information [20,45]. From the perspective of COR theory, this heightened sensitivity to unfairness itself consumes additional cognitive and emotional resources, as individuals must continuously monitor fairness-related cues, regulate negative emotions, and protect themselves from further resource loss [35]. Because EE reflects a state in which individuals’ emotional resources have been excessively depleted [46], these intensified reactions arising from perceptions of unfairness may further drain nurses’ emotional resources and exacerbate EE. Accordingly, the present study proposes the following hypothesis:

**H5.**  *VJS moderates the relationship between ILTAs and EE, such that this relationship will be stronger when VJS is high than when it is low*.

**Figure 1:**
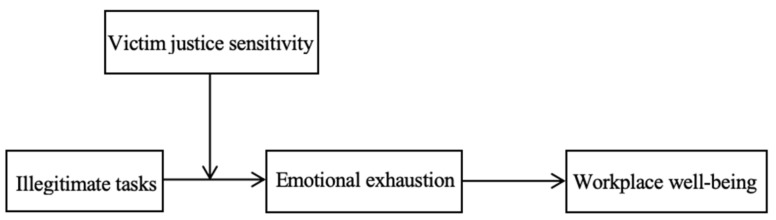
Proposed moderated mediation model.

Taken together, these arguments suggest that the pathway from ILTAs to nurses’ WWB via EE varies with VJS. As discussed above, ILTAs consume nurses’ emotional and psychological resources, thereby increasing EE; in turn, EE further weakens the resources required for nurses to maintain job satisfaction and positive work-related affect. However, this resource depletion process may not be equally strong among all nurses. From the perspective of justice theory, nurses with higher VJS are more likely to interpret ILTAs as unfair and role-devaluing work demands because they are particularly sensitive to whether they are treated unfairly and tend to react more strongly to perceived injustice [21,45]. This justice-related appraisal may intensify the cognitive and emotional effort required to process task-related experiences, regulate negative emotions, and protect their sense of self-worth. According to COR theory, individuals strive to protect their valued resources, and stress arises when these resources are threatened, lost, or when resource investment fails to yield the expected returns [35]. Therefore, when VJS is high, ILTAs are more likely to consume additional cognitive and emotional resources, resulting in greater EE. Furthermore, nurses experiencing EE lack sufficient resources to maintain positive evaluations of their work and favorable work-related affect, thereby reducing their WWB [5,46]. In contrast, when VJS is low, nurses may be less likely to appraise ILTAs as serious threats to justice. Consequently, the resource depletion process through which ILTAs influence WWB via EE is likely to be relatively weaker. Based on this reasoning, the present study proposes the following hypothesis (see Figure 1):

**H6.**  *VJS moderates the mediating relationship of ILTAs and WWB through EE, such that the mediated relationship will be stronger when VJS is high than when it is low*.

## 3. Methods

### 3.1. Study Design

Data were collected in two waves separated by one month. A one-month interval was selected based on both methodological and empirical considerations. Temporal separation between measurement waves has been recommended as an effective approach to reducing common method bias and immediate recall effects [47]. In addition, recent longitudinal research has shown that ILTAs predict stress-related outcomes one month later [48]. Furthermore, because nurses usually have busy schedules and heavy workloads, a more complex multi-wave design could have increased their burden and caused more participants to drop out. Therefore, the two-wave design represented a balance between methodological rigor and practical feasibility, while helping to maintain an adequate sample size and response rate.

We collected data from nurses working in hospitals in different regions of China from March 16 to the end of April 2026. The first wave collected data on ILTAs and VJS. In the second wave, questionnaires were administered to the same participants to measure EE and WWB. To protect the participants’ anonymity while linking responses across the two survey waves, participants were asked to provide the last four digits of their mobile phone number as a matching code. These identifiers were used solely to match responses across the two survey waves and could not be used to identify individual participants. The matching codes were manually checked and verified during the matching process; no duplicate matching codes were identified among the retained questionnaires. Questionnaires that could not be matched across the two survey waves because of participant attrition or invalid responses were excluded from the final analysis. Consequently, the final analytical sample consisted of 497 uniquely matched participants.

### 3.2. Participants and Setting

A non-probability convenience sampling approach was used to recruit the participants. The sample population was recruited from 15 hospitals across 11 cities in China: Beijing, Shanghai, Guangzhou, Shenzhen, Kunming, Zhengzhou, Dalian, Xi’an, Harbin, Zhuhai, and Qingdao. Participating hospitals included tertiary general hospitals, orthopedic specialty hospitals, and traditional Chinese medicine hospitals. Participants were recruited from a wide range of clinical departments, including the emergency department, orthopedics, pediatrics, respiratory medicine, intensive care units (ICUs), psychiatry, cardiovascular medicine, neurology, surgery, and pain management. We used classmates and social networks to establish contact with hospital staff in different regions and, subsequently, contacted head nurses or department directors, who assisted only with questionnaire distribution; they did not participate in monitoring questionnaire completion or have access to the participants’ responses. To ensure voluntary participation and the independence of the responses, several procedures were implemented. Before data collection, the participants were informed of the study objectives and the academic use of their responses through both the informed consent form and verbal instructions. Participation was entirely voluntary, and the participants were informed that they could decline to participate or withdraw from the study at any time without any adverse consequences. All questionnaires were completed independently by the participants. Hospital administrators, head nurses, and department directors did not have access to the individual responses. The questionnaires were completed anonymously, and no names, employee identification numbers, or other directly identifiable personal information was collected. The last four digits of the participants’ mobile phone number were used solely to match responses across the two survey waves and could not be used to identify individual participants. After data collection, electronic data were stored in encrypted files accessible only to members of the research team. Paper questionnaires were stored securely to prevent unauthorized access and protect the participants’ privacy and confidentiality. Eligible participants were required to meet the following conditions: (1) registered nurses, (2) currently employed in a hospital and providing direct patient care, and (3) at least six months of continuous employment in their current hospital. The exclusion criteria included nursing interns, standardized nursing trainees, and administrative personnel who were not directly involved in patient care.

In the first wave of this study, 550 questionnaires were distributed, and 520 were returned. In the second wave, 520 questionnaires were sent out, of which 501 were returned. After the responses were matched based on the participants’ mobile phone number endings, 497 valid cases remained, yielding an effective response rate of 90.36%. This research received ethical approval from the university’s review committee, and each participant gave informed consent before taking part.

The final sample consisted of 497 nurses; the proportion of female participants in the sample was 65.0%, whereas male participants accounted for 35.0%. The relatively high proportion of male nurses may partially reflect the composition of the participating hospitals and clinical departments rather than the recruitment strategy, as the sample included orthopedic specialty hospitals and participants from departments such as orthopedics and intensive care units (ICUs), where male nurses are generally more highly represented than in the nursing workforce overall. Regarding age, participants were most frequently in the 26–35 age range (43.1%), followed by those aged 25 or younger (24.3%) and those aged 36–45 (21.7%). Participants aged 46 or older accounted for the smallest proportion (10.9%). Regarding education, bachelor’s degree holders made up the largest share of the sample (70.2%), while junior college or lower qualifications (17.5%) and master’s degrees or above (12.3%) accounted for smaller proportions. With respect to marital status, 62.6% of the participants were married, 37.0% were single, and 0.4% reported other marital statuses. Regarding work tenure, participants with 0–10 years of work experience accounted for the largest proportion (53.7%), whereas participants whose tenure exceeded 30 years made up the smallest group (4.4%). Table 1 summarizes the sample’s demographic profile.

### 3.3. Measures

The variables in this study were assessed using well-established scales adopted from previous research, which have demonstrated satisfactory reliability and validity. Given the Chinese study context, scales originally developed in English were translated into Chinese using Brislin’s (1980) back-translation procedure [49]. In this study, two bilingual experts were responsible for the Chinese–English translation of the scales. First, the original English items were translated into Chinese, and the Chinese version was then back-translated into English. The research team subsequently compared the back-translated English version with the original English version to identify any semantic discrepancies. If semantic deviations were found between the back-translated version and the original text, the relevant wording in the Chinese translation was revised and refined. This process involved several rounds of translation, back-translation, and revision until the translated version accurately conveyed the meaning of the original scales. Before the formal survey, the research team invited 10 registered nurses to complete the preliminary Chinese questionnaire and provide feedback on item clarity and comprehensibility. Based on their feedback, the research team made minor wording adjustments to items with unclear meanings but did not alter the substantive meaning of the original scales. These nurses were not included in the final analytic sample. The participants rated all items on a five-point Likert scale, from 1 = “strongly disagree” to 5 = “strongly agree”.

Illegitimate Tasks (ILTAs) (Time 1): ILTAs were assessed with the eight-item Bern Illegitimate Tasks Scale (BITS) developed by Semmer et al. (2010) [9]. One example item is “Do you have work tasks to take care of, which you believe should be done by someone else?” In this study, the internal consistency of this scale was α = 0.917.

Victim Justice Sensitivity (VJS) (Time 1): VJS was assessed with the 10-item victim subscale of the Justice Sensitivity Inventory developed by Schmitt et al. (2010) [21]. One example item is “It makes me angry when I am treated worse than others.” In this study, the internal consistency of this scale was α = 0.923.

Emotional Exhaustion (EE) (Time 2): EE was assessed with a 3-item scale adapted from the Maslach Burnout Inventory [46], which was further simplified and applied by Watkins et al. (2015) [22]. One example item is “I feel emotionally drained from my work”. In this study, the internal consistency of this scale was α = 0.898. Although the original MBI emotional exhaustion dimension contains more items, the three-item version has been widely used in organizational and healthcare research because it reduces respondent burden while maintaining satisfactory reliability and construct validity [50,51,52].

Workplace Well-Being (WWB) (Time 2): WWB was measured using the 6-item scale developed by Zheng et al. (2015) [5]. One example item is “I find real enjoyment in my work.” In this study, the internal consistency of this scale was α = 0.907.

Control Variables (Time 1): Based on prior research, gender differences have been associated with variations in burnout dimensions, particularly EE [53]. Age has been found to be associated with individuals’ cognitive evaluations and affective reactions to work contexts [54]. Education level is significantly associated with employees’ job attitudes [54], and organizational tenure has also been shown to be significantly related to individuals’ perceptions and attitudes toward work contexts [55]. In addition, marital status has been closely linked to individuals’ psychological well-being. Prior research suggests that marriage not only enhances subjective well-being but also provides emotional support and social resources that buffer the adverse effects of stress [56,57]. Thus, several demographic characteristics were controlled—including gender, age, education level, organizational tenure, and marital status—to reduce the influence of potential confounding factors on the study’s results.

### 3.4. Data Analysis

The measurement model was evaluated with CFA in Mplus 8.3; descriptive results and bivariate correlations were then calculated in SPSS 27.0. Finally, linear regression analyses were performed in SPSS 27.0, and the hypotheses were examined with the PROCESS v4.0 macro. The 95% confidence intervals were obtained through bootstrapping with 5000 resamples. In addition, a post hoc power analysis was conducted using G*Power 3.1. Assuming a medium effect size (f^2^ = 0.15), a significance level of 0.05, and eight predictors, the achieved statistical power for the final sample (*N* = 497) exceeded 0.99, indicating that the final sample provided adequate statistical power for testing the proposed moderated mediation model.

## 4. Results

### 4.1. Common Method Bias

Because all variables were measured using self-report questionnaires, Harman’s single-factor test was conducted to assess the potential influence of common method bias. The results showed that multiple factors with eigenvalues greater than 1 were extracted, and the first unrotated factor accounted for 39.50% of the total variance, which is below the commonly recommended threshold of 40%; therefore, common method bias was unlikely to pose a serious threat to the findings of this study.

### 4.2. Confirmatory Factor Analysis

To evaluate discriminant validity, a CFA-based comparison was made between the proposed four-factor model and alternative models formed by combining selected constructs (see Table 2). Table 2 reports that the hypothesized four-factor model, in which ILTAs, EE, WWB, and VJS were treated as distinct constructs, demonstrated an acceptable overall fit to the data (χ^2^ = 1168.44, df = 318, χ^2^/df = 3.67, CFI = 0.907, TLI = 0.897, RMSEA = 0.073, SRMR = 0.046) and outperformed the alternative models. These results suggest that the four core constructs can be empirically distinguished from one another, providing acceptable support for the discriminant validity of ILTAs, EE, WWB, and VJS.

In addition, composite reliability (CR) and average variance extracted (AVE) were calculated to further assess construct reliability and convergent validity. As shown in Table 3, the standardized factor loadings ranged from 0.670 to 0.912, Cronbach’s α coefficients ranged from 0.898 to 0.923, CR values ranged from 0.901 to 0.924, and AVE values ranged from 0.548 to 0.753; these results provide additional support for satisfactory internal consistency, construct reliability, and convergent validity.

### 4.3. Descriptive Statistics and Correlations

The descriptive statistics and correlations for the main variables are reported in Table 4. As shown in the table, EE was higher among those reporting more ILTAs (r = 0.512, *p* < 0.001) and VJS (r = 0.391, *p* < 0.001), as well as lower WWB (r = −0.459, *p* < 0.001). Moreover, higher EE corresponded to lower WWB (r = −0.594, *p* < 0.001) and was positively correlated with VJS (r = 0.485, *p* < 0.001). VJS was also negatively associated with WWB (r = −0.437, *p* < 0.001). Overall, the results align with the expected directions of the hypotheses, offering an initial basis for further examination of the mediation and moderation effects. In addition, variance inflation factors (VIFs) were checked to evaluate potential multicollinearity. Although age and work tenure were highly correlated, the VIF results remained within the acceptable range, with the maximum value being 4.712, below the recommended cutoff of 5.0. These results suggest that multicollinearity was unlikely to affect the present analyses substantially.

### 4.4. Hypothesis Testing

Model 4 in Table 5 shows that nurses reporting more ILTAs tended to have lower WWB (B = −0.359, *p* < 0.001); H1 is therefore supported, indicating that nurses who reported more ILTAs tended to have lower WWB. Model 2 in Table 5 further shows that ILTAs are positively linked to higher EE (B = 0.599, *p* < 0.001); this finding supports H2, suggesting that ILTAs are positively associated with nurses’ EE. Model 5 shows that nurses with higher EE tended to report lower WWB (B = −0.383, *p* < 0.001), providing support for H3, which proposes a negative association between EE and WWB.

The mediating effect of EE was examined using PROCESS Model 4 with 5000 bootstrap samples. As shown in Table 6, the indirect effect of ILTAs on WWB through EE was statistically significant (Effect = −0.186, Boot SE = 0.026, 95% CI [−0.239, −0.137]), as the bootstrap confidence interval did not include zero. These findings indicate a significant indirect effect of ILTAs on WWB through EE, thereby supporting Hypothesis 4.

**Figure 2:**
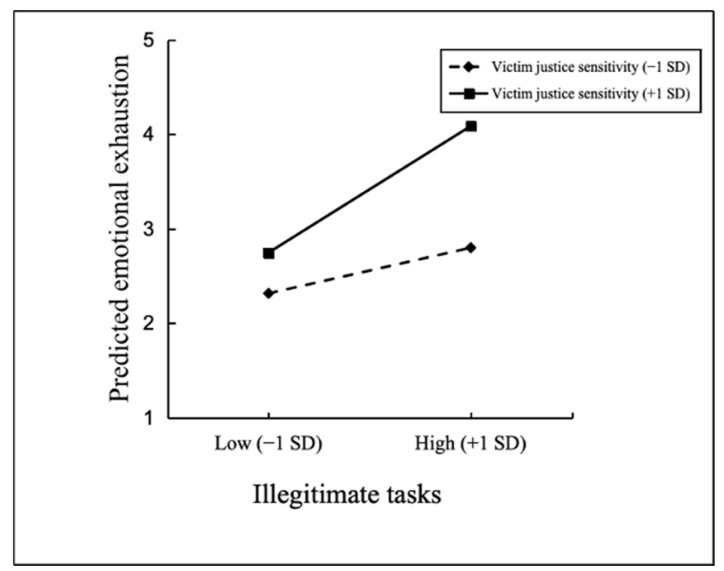
Moderation of the ILTAs–EE link by VJS.

The moderation hypothesis was examined using PROCESS Model 7. Prior to constructing the interaction term, ILTAs and VJS were standardized (z-score transformed), and the interaction term was computed by multiplying the standardized variables. As shown in Model 6 of Table 5, the interaction between ILTAs and VJS was significantly associated with EE (B = 0.214, *p* < 0.001), indicating a significant moderation effect and supporting Hypothesis 5. To facilitate interpretation of the interaction effect, simple slope analyses were conducted at one standard deviation above and below the mean of VJS. As illustrated in Figure 2, the positive association between ILTAs and EE was significant at both low (−1 SD) and high (+1 SD) levels of VJS but was substantially stronger among nurses with high VJS. Specifically, when VJS was low, the association between ILTAs and EE was weaker (B = 0.255, SE = 0.058, 95% CI [0.141, 0.368], *p* < 0.001); in contrast, when VJS was high, the effect was substantially stronger (B = 0.706, SE = 0.061, 95% CI [0.586, 0.827], *p* < 0.001). Furthermore, the Johnson-Neyman analysis indicated that the association between ILTAs and EE became statistically significant when VJS exceeded −1.176 standard deviations from the mean, providing additional evidence for the robustness of the moderation effect.

Next, this study used the SPSS PROCESS with 5000 bootstrap samples to test H6. As reported in Table 7, the moderated mediation index was significant (Index = −0.090, 95% CI [−0.121, −0.060]), and the 95% confidence interval did not include zero. This result lends evidence to the proposed moderated mediation model—specifically, the indirect pathway linking ILTAs to nurses’ WWB via EE became stronger at higher levels of VJS and weaker at lower levels; thus, the data support H6.

## 5. Discussion

Guided by COR theory and justice theory, the present findings provide evidence consistent with the proposed relationships among ILTAs, EE, WWB, and VJS in the nursing context.

Firstly, the negative association between ILTAs and WWB echoes previous research suggesting that ILTAs deviate from individuals’ professional roles and may be perceived as unreasonable or unnecessary work arrangements. Such tasks may threaten occupational identity and thereby undermine individuals’ work-related affective experiences [16]. Building on this, the present findings further suggest that such tasks may place extra demands on nurses while disrupting how they understand their professional roles, making it harder for them to gain the expected sense of accomplishment and meaning from daily practice [28]. At the same time, repeated exposure to ILTAs may lead nurses to feel that their work is not respected or properly valued, thereby lowering their overall evaluation of work. In addition, such tasks may give rise to adverse feelings, including anxiety, irritation, and discouragement [36], which can further undermine individuals’ positive emotional experiences and job satisfaction [58].

Secondly, the results suggest that EE is one pathway linking ILTAs to lower WWB among nurses. Previous studies have linked ILTAs to higher EE, and this form of strain has also been associated with poorer health and well-being outcomes, suggesting that it may serve as a key mediator in this relationship [59]. Building on this, the present findings help explain one possible mechanism underlying this relationship. Based on COR theory, ILTAs compel nurses to devote substantial personal resources such as time, effort, and attention to work duties that go beyond expected professional requirements [16], thereby weakening their ability to maintain existing resources. When resource investment remains mismatched with returns over time, individuals may experience greater resource depletion [35,36], which may ultimately lead to EE among nurses. When emotionally exhausted, individuals lack both the willingness and the capacity to continue investing resources [60,61], thereby reducing their overall evaluation of work and, ultimately, undermining their WWB; consequently, they are less likely to experience positive work-related affect or job satisfaction, providing evidence consistent with EE serving as an important explanatory pathway linking ILTAs to WWB.

Thirdly, our findings show that nurses’ level of VJS changes how strongly ILTAs are associated with EE. Prior research suggests that unfair treatment elicits stronger negative emotional responses among individuals high in VJS [21]. Building on this, the present study draws on COR theory and justice theory to further explain this moderating mechanism. Justice theory suggests that ILTAs may be viewed by individuals as unfair and inappropriate work demands [16]. For nurses with higher VJS, ILTAs may be interpreted not only as additional work demands but also as a form of unfair treatment. From the perspective of the COR theory, these amplified perceptions of unfairness may intensify individuals’ subjective appraisal of resource loss and accelerate the process of resource depletion, thereby being associated with higher levels of EE [21,26]. Accordingly, the present findings provide evidence consistent with VJS functioning as an important boundary factor shaping the strength of the ILTAs–EE relationship.

Finally, these findings need to be understood in light of China’s cultural background, where power distance tends to be relatively high. In such contexts, employees are usually less likely to question or refuse tasks given by their supervisors, even when these tasks are not part of their formal job duties [19]; consequently, nurses may be more inclined to perceive ILTAs as unavoidable job demands that consume valuable personal resources. In contrast, employees in lower-power-distance cultures may possess greater autonomy to question or reject role-incongruent tasks, potentially weakening the relationships observed in the present study; therefore, caution should be exercised when generalizing the findings beyond similar cultural and healthcare contexts.

### 5.1. Theoretical Implications

This research contributes to the literature in several ways:

First, earlier COR-based studies have largely conceptualized work stress in terms of general stressors and have primarily focused on their negative outcomes, giving less consideration to the distinct processes by which different types of stressors affect employees. Moreover, such research has mostly been conducted in general work contexts, thereby overlooking contextual differences in how stressors function within specific occupational settings. In particular, in the nursing context, nursing work is characterized by high intensity and substantial emotional labor [62,63], making stressors more likely to exert significant effects on individuals’ emotions [64]. Accordingly, this study conceptualizes ILTAs as a specific stressor that violates role expectations and threatens individuals’ occupational identity [9,33]; it suggests that such tasks may contribute to EE through resource loss and, in turn, reduce nurses’ WWB. In this way, the present study moves beyond prior studies that have mainly emphasized general stressors and offers a deeper understanding of how distinct stressor types shape WWB.

Second, existing research based on the COR theory has mostly emphasized the direct effects of resource loss on individuals, while relatively overlooking the cognitive appraisal process through which individuals respond to stress. In nursing work, ILTAs often imply the disruption of role boundaries and may be perceived by individuals as unfair work arrangements, thereby eliciting perceptions of injustice [16]. Based on justice theory, we propose that individuals’ subjective perceptions of unfair situations influence their emotional reactions [65,66,67] and play a key role in the stress response process [68]. Specifically, VJS, as a personal trait, may amplify individuals’ interpretations of unfairness [21,69], thereby eliciting stronger negative emotional reactions and contributing to higher EE through the continuous depletion of emotional resources. Therefore, the present findings extend previous research by providing evidence consistent with an amplification pattern in the stressor-response process within the nursing context, whereby perceptions of unfairness may strengthen the association between ILTAs and EE.

Finally, prior research has often relied on a single theoretical perspective to explain the role of stressors in shaping individual responses, with limited attention to multi-theoretical integration. By integrating COR theory and justice theory, the present study adopts the dual perspectives of resource loss and perceived unfairness to further reveal how cognitive appraisal is embedded in the process of resource depletion, thereby providing an integrated theoretical perspective for understanding the pathways through which stressors influence individual outcomes.

### 5.2. Practical Implications

This study provides three practical suggestions for healthcare institutions aiming to improve nurses’ WWB:

First, healthcare institutions should reduce the assignment of ILTAs to nurses. The COR theory posits that alleviating stress should not rely solely on individual-level adjustments but, rather, on reducing sources of resource depletion at the institutional and environmental levels [60]. Therefore, hospital managers can improve work processes and make nurses’ job responsibilities clearer. For example, nurses should not often be asked to do administrative work that is not related to professional nursing care, such as clerical work, documentation, inventory management, and data reporting. Instead, these non-nursing tasks should be given to suitable support staff to reduce ILTAs. In addition, healthcare institutions may establish support mechanisms, such as a reserve nursing workforce or temporary support teams, to assist departments experiencing heavy workloads, thereby reducing the need to assign non-core duties to frontline nurses. Furthermore, for nurses who frequently undertake temporary, unexpected, or non-core tasks, healthcare institutions could enhance their sense of recognition and appreciation by providing performance-based bonuses, overtime compensation, compensatory leave, flexible scheduling, public recognition, professional development opportunities, and career advancement support.

Second, hospital managers should notice changes in nurses’ emotions and provide timely support [70]. This study suggests that EE is an important pathway through which ILTAs are associated with lower WWB among nurses. Previous research has shown that psychological support, stress management training, and organizational interventions can help reduce EE and work-related stress [71,72]; thus, hospital managers can take targeted measures either before EE develops or after it has already emerged, so as to reduce the pressure associated with ILTAs. At the organizational level, healthcare institutions may adopt flexible scheduling, ensure sufficient rest time, and optimize staffing arrangements to help alleviate work pressure and facilitate psychological recovery. At the team level, regular team-building activities and peer support programs may help nurses share work-related stress, strengthen emotional resilience, and enhance mutual support among colleagues. At the individual level, nurses can be provided with psychological counseling and stress management training to strengthen their coping abilities and reduce EE.

Finally, healthcare institutions should enhance fairness and transparency in the task allocation process from the multidimensional perspective of justice theory. According to justice theory, individuals evaluate whether they are treated fairly within an organization by considering whether distributive outcomes are reasonable, whether decision-making procedures are fair, and whether they receive adequate explanations, dignity, and respect during interpersonal interactions in the implementation of decisions [25]. Specifically, in terms of distributive justice, managers should ensure that task allocation is aligned with nurses’ professional roles and competencies; for example, tasks should be allocated equitably according to nurses’ qualifications and experience, departmental workload, patient care needs, and shift schedules. In terms of procedural justice, healthcare institutions should make task allocation more open, transparent, and consistent; they should also clarify the criteria used in assigning tasks and give nurses opportunities to express their views or take part in relevant decisions. In this way, nurses may be more likely to understand and accept the decision-making process. For example, hospitals may establish written policies specifying which non-core duties may be assigned to nurses, under what circumstances such assignments are appropriate, and who is responsible for approving these assignments. In terms of interactional justice, managers should communicate with nurses in a respectful and considerate manner when assigning tasks. When task arrangements are necessary, they should provide clear explanations and address nurses’ concerns, which may help reduce nurses’ feelings of being disrespected or ignored.

## 6. Conclusions

This study adds to existing research on ILTAs by further looking at how ILTAs are related to nurses’ WWB. By integrating COR theory and justice theory, it provides a more comprehensive explanation of how and under what conditions ILTAs are associated with nurses’ WWB. Specifically, COR theory explains the resource depletion process through EE, whereas justice theory explains why this process is more pronounced among nurses with higher VJS. The findings indicate that ILTAs are associated with lower WWB both directly and indirectly through EE and that VJS strengthens the association between ILTAs and EE, suggesting that nurses who are more sensitive to unfair treatment may experience greater emotional strain when they have to do tasks that are not part of their nursing role. These findings highlight the need for healthcare institutions to reduce unnecessary and unreasonable task assignments, improve fairness and transparency in task allocation, and provide timely emotional support for nurses. Such efforts may help protect nurses’ WWB and improve the quality and sustainability of healthcare services. Future research may further examine the proposed model using longitudinal or cross-cultural designs and explore additional protective factors, such as psychological resilience and positive organizational resources, in order to further advance our understanding of how ILTAs are associated with nurses’ WWB.

## 7. Limitations and Future Directions

The present study is subject to several limitations:

First, although the two-wave design helped alleviate common method bias by introducing temporal separation, the main variables were still measured through self-reports. Moreover, EE and WWB were measured at the same timepoint (Time 2), limiting our ability to draw strong temporal or causal inferences regarding the proposed mediation process. Future longitudinal studies with three or more measurement waves will be needed to provide stronger evidence regarding the temporal ordering of the proposed mediation mechanism. In addition, future studies could draw on data provided by different respondent groups, including supervisors and colleagues, to further alleviate potential common method bias.

Second, we recruited participants from hospitals in several Chinese cities using convenience sampling. Although the sample included nurses from multiple hospitals and departments, the non-probability sampling strategy may have limited the representativeness of the sample. Future studies may employ random sampling techniques and recruit participants from a broader range of healthcare settings in order to enhance the generalizability of the findings.

Third, this study was situated in the healthcare setting of China. Under the influence of different cultural values, nurses may differ in their perceptions of and attitudes toward ILTAs; therefore, caution should be exercised when generalizing the findings to other cultural contexts or occupational groups. Future cross-cultural research is needed to determine whether the proposed relationships hold across diverse cultural and healthcare contexts.

Finally, the analysis centered on work stressors related to nurses’ well-being at work and considered the ways in which unfavorable work conditions shape their mental states and behavioral responses. However, emerging research suggests that a purely deficit-oriented perspective may be insufficient to fully explain the formation mechanism of WWB. Future research should therefore further shift attention toward positive promoting factors. Specifically, research should focus on identifying which organizational and individual factors can promote nurses’ positive emotions, work engagement, interpersonal relationships, sense of meaning, and sense of accomplishment, thereby enabling them to “flourish” in their work [73]. Therefore, future research on the determinants of WWB among nurses could prioritize positive aspects, thereby complementing and expanding existing stress-centered research frameworks. Psychological resilience represents nurses’ ability to manage stressful circumstances and reduces the negative influence of stressors on them [74,75]. Subsequent studies may examine psychological resilience to clarify when and for whom ILTAs are most likely to influence nurses.

## Figures and Tables

**Table 1:** Study sample characteristics (*N* = 497).

Sample Characteristics	*n*	%
Gender		
Female	323	65.0%
Male	174	35.0%
Age		
25 years or below	121	24.3%
26–35 years	214	43.1%
36–45 years	108	21.7%
46 years or above	54	10.9%
Education		
Junior college or below	87	17.5%
Bachelor’s degree	349	70.2%
Master’s degree or above	61	12.3%
Marital status		
Married	311	62.6%
Single	184	37.0%
Others	2	0.4%
Work tenure (years)		
0–10	267	53.7%
11–20	140	28.2%
21–30	68	13.7%
>30	22	4.4%

Note: Education and work tenure are presented in grouped categories for descriptive purposes only. In subsequent analyses, education was entered using its original coding scheme, whereas work tenure was entered as the actual number of years worked.

**Table 2:** Results of confirmatory factor analysis.

Model	χ^2^	df	χ^2^/df	CFI	TLI	RMSEA	SRMR
Four-factor model (ILTAs, EE, WWB, VJS)	1168.44	318	3.67	0.907	0.897	0.073	0.046
Three-factor model (ILTAs + EE, WWB, VJS)	1935.80	321	6.03	0.822	0.806	0.101	0.083
Two-factor model (ILTAs + WWB, EE + VJS)	3264.36	323	10.11	0.677	0.649	0.135	0.123
One-factor model (ILTAs + EE + WWB + VJS)	4549.45	324	14.04	0.535	0.497	0.162	0.131

Note: *N* = 497; ILTAs: illegitimate tasks; EE: emotional exhaustion; VJS: victim justice sensitivity; WWB: workplace well-being.

**Table 3:** Reliability and convergent validity.

Construct	Standardized Factor Loadings	Cronbach’s α	CR	AVE
ILTAs	0.683–0.856	0.917	0.917	0.583
EE	0.836–0.912	0.898	0.901	0.753
WWB	0.702–0.850	0.907	0.909	0.625
VJS	0.670–0.807	0.923	0.924	0.548

Note: *N* = 497; ILTAs: illegitimate tasks; EE: emotional exhaustion; VJS: victim justice sensitivity; WWB: workplace well-being.

**Table 4:** Means, SDs, and correlations.

Variable	M	SD	1	2	3	4	5	6	7	8
1 Gender	0.350	0.477								
2 Age	2.190	0.928	0.126 **							
3 Education	4.880	0.706	0.008	−0.055						
4 Marriage	1.380	0.494	−0.110 *	−0.656 ***	−0.035					
5 Work tenure	10.390	9.006	0.075	0.859 ***	−0.189 ***	−0.575 ***				
6 ILTAs	3.110	0.947	−0.052	−0.064	−0.013	0.119 **	−0.028			
7 EE	3.070	1.162	−0.047	−0.220 ***	−0.045	0.251 ***	−0.146 **	0.512 ***		
8 WWB	3.496	0.781	0.014	0.161 ***	0.006	−0.224 ***	0.074	−0.459 ***	−0.594 ***	
9 VJS	3.073	0.779	−0.064	−0.157 ***	−0.077	0.233 ***	−0.078	0.391 ***	0.485 ***	−0.437 ***

Note: *N* = 497; * *p* < 0.05; ** *p* < 0.01; *** *p* < 0.001; ILTAs: illegitimate tasks; EE: emotional exhaustion; VJS: victim justice sensitivity; WWB: workplace well-being; Gender was coded as 0 = female and 1 = male; Age was coded as 1 = 25 years or below, 2 = 26–35 years, 3 = 36–45 years, and 4 = 46 years or above; Education was coded as 1 = primary school or below, 2 = junior high school, 3 = high school/technical secondary school, 4 = junior college, 5 = bachelor’s degree, and 6 = postgraduate degree or above; Marital status was coded as 1 = married, 2 = single, and 3 = others; Work tenure was measured as the actual number of years worked.

**Table 5:** Regression results.

	EE			WWB		
Variable	M1	M2	M3	M4	M5	M6
Constant	3.062 ***	1.424 **	3.981 ***	4.962 ***	5.153 ***	3.246 ***
Gender	−0.021	0.023	−0.035	−0.062	−0.043	0.056
Age	−0.289 *	−0.252 *	0.215 **	0.193 **	0.105	−0.174
Education	−0.035	−0.042	−0.044	−0.040	−0.057	−0.037
Marriage	0.443 **	0.286 *	−0.348 ***	−0.254 **	−0.179 *	0.174
Work tenure	0.020	0.013	−0.024 **	−0.020 **	−0.016 *	0.005
ILTAs		0.599 ***		−0.359 ***		0.455 ***
EE					−0.383 ***	
VJS						0.430 ***
ILTAs × VJS						0.214 ***
R^2^	0.076	0.310	0.069	0.255	0.369	0.425
ΔR^2^		0.234		0.185	0.299	0.042
F	8.120 ***	36.707 ***	7.311 ***	27.908 ***	47.713 ***	45.112 ***

Note: *N* = 497; * *p* < 0.05; ** *p* < 0.01; *** *p* < 0.001; ILTAs: illegitimate tasks; EE: emotional exhaustion; VJS: victim justice sensitivity; WWB: workplace well-being.

**Table 6:** Bootstrapping mediation test results.

Path	Effect Type	Effect	SE	LLCI	ULCI
ILTAs → WWB	Total effect	−0.359	0.033	−0.423	−0.295
ILTAs → WWB	Direct effect	−0.173	0.034	−0.239	−0.107
ILTAs → EE → WWB	Indirect effect	−0.186	0.026	−0.239	−0.137

Note: *N* = 497; ILTAs: illegitimate tasks; EE: emotional exhaustion; WWB: workplace well-being; SE: standard error; LLCI: lower limit of confidence interval; ULCI: upper limit of confidence interval.

**Table 7:** Moderated mediation analysis results.

			95% Confidence Interval	
Moderator	Effect	SE	LLCI	ULCI
Low VJS (−1 SD)	−0.079	0.021	−0.124	−0.040
High VJS (+1 SD)	−0.219	0.029	−0.279	−0.164
Moderated Mediation Effect Index	−0.090	0.016	−0.121	−0.060

Note: VJS: victim justice sensitivity; LLCI: lower limit of confidence interval; ULCI: upper limit of confidence interval.

## Data Availability

The data presented in this study are available upon request from the corresponding author. The data are not publicly available due to privacy and ethical restrictions.
